# Marked platelet transfusion refractoriness caused by anti-HLA-I and anti-HPA-5b antibodies: A case report

**DOI:** 10.1097/MD.0000000000044945

**Published:** 2025-10-24

**Authors:** Yihan Wang, Zhen Liu, Shengbao Duan, Chunyan Lin, Yiming Jin, Hongmei Wang, Yujue Wang, Longhai Tang, Mingyuan Wang

**Affiliations:** a Suzhou Blood Center, Suzhou, Jiangsu, China; b Suzhou Institute of Biomedical Engineering and Technology, Chinese Academy of Sciences, Suzhou, Jiangsu, China, China; c Department of Blood Transfusion, the First Affiliated Hospital of Soochow University, Suzhou, Jiangsu, China.

**Keywords:** antibody analysis, HLA class I, HPA-5b, platelet antibody, platelet transfusion refractoriness

## Abstract

**Rationale:**

Platelet transfusions are the key treatment for patients with severely low platelet counts and significant bleeding symptoms. Platelet transfusion refractorines (PTR) is defined as unsatisfactory transfusion of platelets with at least 2 ABO blood groups matched and stored for <72 hours.

**Patient concerns:**

A 37-year-old woman presented for medical evaluation due to the cessation of fetal heart activity after 50 days of gestation.

**Diagnoses:**

AML-M2, PTR.

**Interventions:**

To explore potential immune-mediated causes of platelet refractoriness, the following diagnostic evaluations were conducted: Platelet antibody screening using the PAK-Lx assay (Immucor) to detect anti-HLA class I and HPA antibodies. Anti-HLA class I specificity determination via LIFECODES LSA Single Antigen bead assays (Luminex). Molecular typing of HLA class I and HPA alleles using PCR-sequence based typing (PCR-SBT), as well as CD36 phenotyping via flow cytometry. Confirmation testing for anti-HPA-5b antibodies using chloroquine-treated platelet solid-phase assays.

**Outcomes:**

The patient had high-fluorescence-intensity HLA antibodies along with HPA antibodies, resulting in severe refractoriness to platelet transfusion.

**Lessons:**

Various methods were used to comprehensively analyze the specificity of anti-platelet antibodies in the samples of patients with ineffective platelet transfusion, thus providing a reference for the clinical diagnosis and treatment of PTR.

## 1. Introduction

Platelet transfusion refractorines (PTR) is a serious complication caused by the development of anti-platelet antibodies after multiple platelet transfusions. Both immune and nonimmune factors can lead to PTR. In addition to nonimmune factors, such as fever, splenomegaly, and low platelet quality, various immune factors, such as the anti-HLA, anti-HPA, and anti-CD36 antibodies, also mediate PTR.^[1]^ Multiple pregnancies significantly increase the risk of alloimmunity due to fetal-maternal HLA incompatibility.^[2]^ Platelet refractoriness is also caused by anti-HLA and anti-HPA-5a alloantibodies during clinical allogeneic hematopoietic stem cell transplantation.^[3]^ Here, we present a case of Acute Myeloid Leukemia (AML) with severe PTR associated with anti-HLA-I and anti-HPA-5b antibodies that was successfully treated via compatible platelet transfusion.

## 2. Case report

The patient was a 32-year-old woman with a history of multiple pregnancies (obstetric history: G5 T2). During the management of fetal demise at 50 days of gestation, she was diagnosed with AML-M2 leukemia. The patient received 8 platelet transfusions within a 14-day period (total dose: 20 × 10^11^ platelets). Despite an adequate volume of blood transfusions, her platelet count did not show a sustained increase, and her clinical bleeding symptoms remained unimproved. The physical examinations and blood tests conducted upon patient admission can effectively rule out nonimmune causes such as fever and disseminated intravascular coagulation. Next, let’s focus on the detection of immune factors.

### 2.1. Reagents and instruments

TGuide S32 Magnetic Blood DNA kit 3 (No. KJLTY1313; TianGen Biotech (Beijing) Co., Ltd., China). Human Platelet-Specific Antigen Genotyping Detection Kit (Lot No.: 202110a; Jiangsu Weihe Biotechnology Inc., Jiangsu, China), PAK-Lx kit (Lot No.: 3012631; Immucor, Beijing Bofurui Gene Diagnostic Technology Co., Ltd., Beijing, China), and LIFECODES LSA Single Antigen kit (batch number: 3012154; Immucor) were used in this study. Additionally, solid-phase immunosorbent reagent for platelet antibody detection (batch number: 20230302; Suzhou Institute of Biomedical Engineering and Technology, Chinese Academy of Sciences, Suzhou, Jiangsu, China), chloroquine diphosphate (Merck Company, Darmstadt, Germany), phycoerythrin (PE)-labeled anti-human CD36 monoclonal antibody (batch number: B286438; Biolegend, San Diego), PE-labeled anti-human IgG antibody (batch number: 403503; Biolegend), 3730 gene sequencer (Applied Biosystems), CFX96 deep well real-time polymerase chain reaction (PCR) instrument (Bio-Rad), and Luminex FLEXMAP 3D instrument (Thermo Fisher Scientific, Waltham) were used in this study.

### 2.2. Platelet antibody detection and analysis

Patient plasma was incubated with the PAK-Lx microbeads coated with different specific antigens (HLA-I, GPIIb/IIIa, GPIb/IX, GPIa/IIa, and CD36) at room temperature (20°C–25°C), according to the instructions of the PAK-Lx Assay kit. The unbound plasma components were washed and removed, and a PE-labeled anti-human IgG antibody was added. After incubation at room temperature, the reaction mixture was diluted and analyzed using the Luminex instrument. Then, mean fluorescence intensity (MFI) of the beads was compared with that of the negative control to determine whether the specimen contained any platelet-specific antibodies (MFI < 750: negative; MFI ≥ 750: positive).

### 2.3. Detection of anti-HLA-I antibody specificity

Anti-HLA-I antibody was detected using the fluorescence flow Luminex technology according to the instructions of the LIFECODES LSA Single Antigen Kit. Patient samples and HLA-I antigen-coated microbeads (containing 97 specific HLA-A, -B, and -C antigens) were added to the Millipore microplates. After incubation, if the sample contained an anti-HLA-I antibody, it bound to the antigen on the microbeads. After incubation, MFI of specific binding beads was determined using the Luminex platform (MFI ≤ 5000: negative, 5000 < MFI ≤ 10,000: weak positive, 10,000 < MFI ≤ 15,000: medium positive, and MFI > 15,000: positive) and then the specific antigen type was obtained via software analysis.

Based on the platelet antibody detection results, HPA-5aa and HPA-5ab type O platelets were collected, treated with 0.2 M (pH 4.0) chloroquine diphosphate to remove the HLA-I antigen, and analyzed via a solid-phase immunosorbent assay. Positive samples containing anti-HLA-I antibodies were used as controls. Briefly, 50 µL of the appropriate concentration of HPA-5aa and HPA-5ab type O platelets were added to the reaction plate and centrifuged at 50 × g for 5 minutes. After washing with phosphate-buffered saline containing 0.05% Tween 20 (PBST), 100 µL of 0.2 M (pH 4.0) chloroquine diphosphate was added to the treated wells, physiological saline was added to the untreated wells, and incubated at room temperature for 30 minutes. After the addition of the low ionic strength saline solution (100 µL), patient plasma and control specimens were washed, incubated at 37°C for 30 minutes, mixed with 50 µL of anti-human IgG and indicator red cells, and centrifuged. Presence of combined red blood cells at the bottom of the well indicated a positive reaction and formation of a red blood cell layer at the bottom of the well indicated a negative reaction.

### 2.4. HLA and HPA typing

DNA was extracted from the patient sample and subjected to HLA and HPA typing via PCR. The result file of the PCR amplification instrument was imported into the Immuno Gene Analyzer interpretation software and analyzed.

### 2.5. Platelet surface CD36 antigen detection

Platelet surface CD36 expression was determined via a fluorescence assay using a specific anti-CD36 monoclonal antibody. Briefly, 50 μL platelet-rich plasma of the patient was put into a flow cytometer tube and mixed with 5 μL PE fluorescently labeled anti-human CD36 monoclonal antibody, and PE fluorescently labeled anti-human IgG antibody was used as an isotype control. After thorough mixing, the mixture was incubated at 37°C in the dark for 20 minutes. After incubation, platelets were centrifuged at 1200 × g for 5 minutes, and the supernatant was discarded and suspended in 400 μL of PBS. Then, MFI on the platelet surface was measured via flow cytometry.

### 2.6. The corrected count of increment (CCI)

CCI serves as the primary index for assessing the efficacy of platelet transfusion. When a patient receives fresh platelets of the same ABO type, if the 1-hour CCI is <7.5 × 10^9^/L and the 18 to 24-hour CCI is <4.5 × 10^9^/L, the platelet transfusion is deemed ineffective. The calculation formula for CCI is as follows: CCI = (post-infusion Plt − pre-infusion Plt) (10^9^/L) × body surface area (m²) ÷ total number of small blood transfusion plates (10¹¹). (Note: Body surface area can be calculated using the formula: BSA = 0.0061 × height + 0.0128 × weight − 0.01529). Due to challenges in obtaining the 1-hour CCI in actual clinical practice, this study utilized the 24 hour CCI instead. Complete Blood Count Analysis.

## 3. Results

### 3.1. Platelet antibody detection

Initial glycoprotein antibody profiling revealed complex immunoreactivity patterns in Table 1.

**Table 1:** Platelet glycoprotein antibody reactivity.

Target glycoprotein	Reactivity	Interpretation
Pan-glycoprotein	Strong positive	Anti-HLA-I antibodies present
GPIa/IIa	Weak positive	Suspect anti-HPA antibodies
GPIIb/IIIa	Negative	Rule out common alloantibodies
GPIb/IX	Negative	
CD36	Negative	Exclude CD36 deficiency

### 3.2. Specificity of anti-HLA-I antibodies

**Figure 1.**
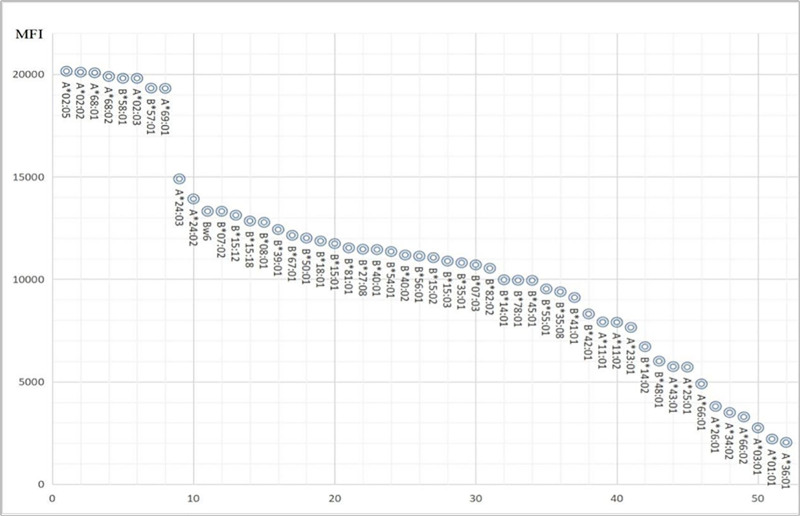
Specific test results of anti-HLA class I antibody.

PRA positivity reached 54% across HLA-A/B/C loci. Antibody strength distribution and corresponding alleles are systematically categorized in Table 2 (Fig. 1).

**Table 2:** HLA-I antibody strength stratification.

Reactivity group	HLA alleles identified	Clinical relevance
Strong (+++)	A*02:05, A*02:02, A*68:01, A*68:02, B*58:01, A*02:03, B*57:01, A*69:01	High transfusion refractoriness risk
Medium (++)	A*24:03, A*24:02, Bw6, B*07:02, B*15:12, B*15:18, B*08:01, B*39:01, B*67:01, B*50:01	Moderate clinical impact
Weak (+)	B*14:01, B*78:01, B*45:01, B*55:01, B*35:08, B*41:01	Potential cross-reactivity
Negative (−)	A*66:01, A*26:01, A*34:02, A*66:02, A*03:0*	Non-reactive alleles

Strength defined by MFI thresholds: strong > 15,000; medium 10,000–15,000; weak 5000–10,000 (per LABScreen® criteria).

### 3.3. Specificity of anti-HPA antibodies

**Figure 2.**
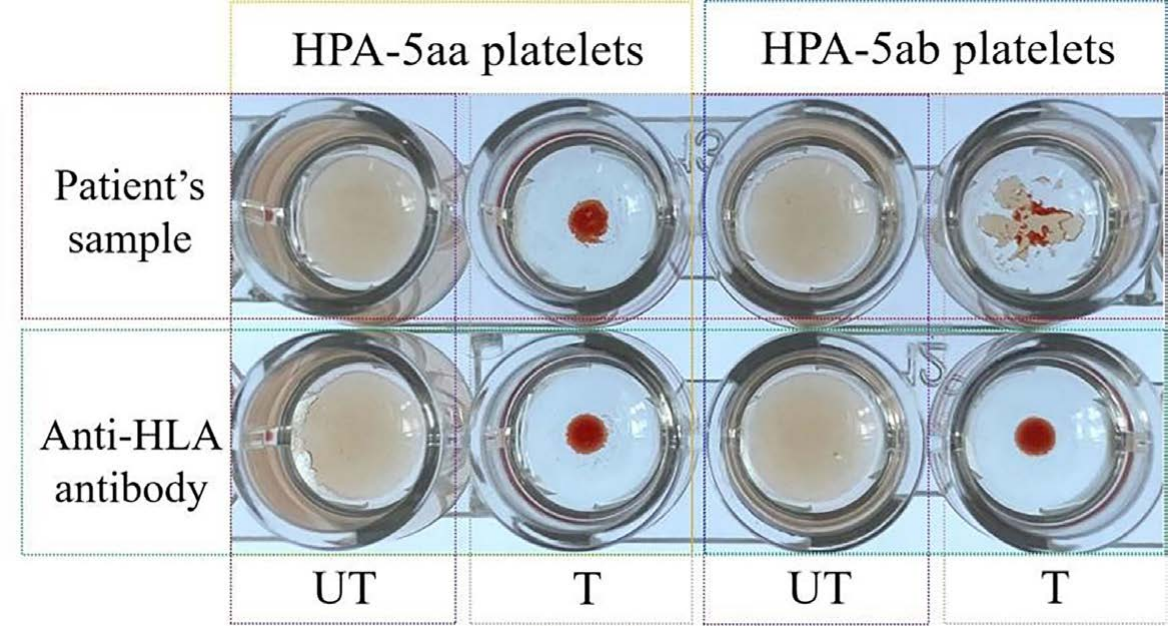
Specific detection results of anti-HPA-5b antibody. T = treated with chloroquine diphosphate; UT = not treated with chloroquine diphosphate.

Chloroquine stripping assays on HPA-5aa/5ab platelets confirmed anti-HPA-5b specificity, with reactivity patterns detailed in Table 3. MAIPA validation further corroborated these findings (Fig. 2).

**Table 3:** HPA-5 antibody verification assay.

Platelet antigen used	Test condition	Reaction result	Interpretation
HPA-5aa	Untreated platelets	Positive	Initial reactivity
	Chloroquine-treated platelets	Negative	Reactivity removed (HLA-dependent)
HPA-5ab	Untreated platelets	Positive	Initial reactivity
	Chloroquine-treated platelets	Weak positive	Residual reactivity (HPA-5b specific)
Control (Anti-HLA-I)	Untreated platelets	Positive	Initial reactivity
	Chloroquine-treated platelets	Negative	Validates HLA removal
MAIPA confirmation			Presence of anti-HPA-5b antibodie

### 3.4. HLA and HPA genotyping and CD36 detection

**Figure 3.**
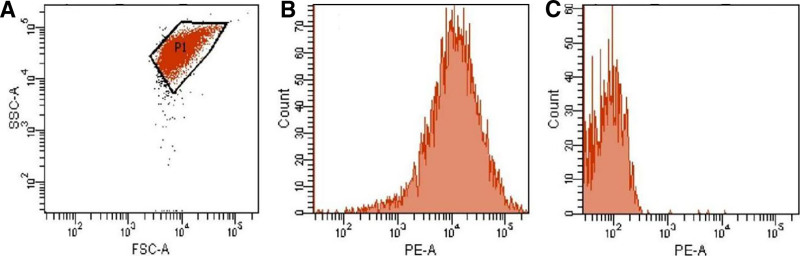
Platelet CD36 test results of patient. (A) Platelet circle gate; (B) patient platelet CD36 detection; (C) isotype negative control.

Genetic profiling and antigen expression results in Table 4 (Fig. 3).

**Table 4:** Patient genotype and phenotype profile.

Category	Specific results
HLA-I Genotype	A*30:01, A*33:03 B*13:02, B*44:03 C*06:02, C*14:03
HPA Genotype	HPA-1aa/2aa/3ab/4aa/5aa/6aa/10aa/15ab/21aa *(HPA-5 homozygous aa)*
CD36 Expression	Normally expressed on platelet surface (Confirmed by flow cytometry)

### 3.5. Compatible platelet transfusion

**Figure 4.**
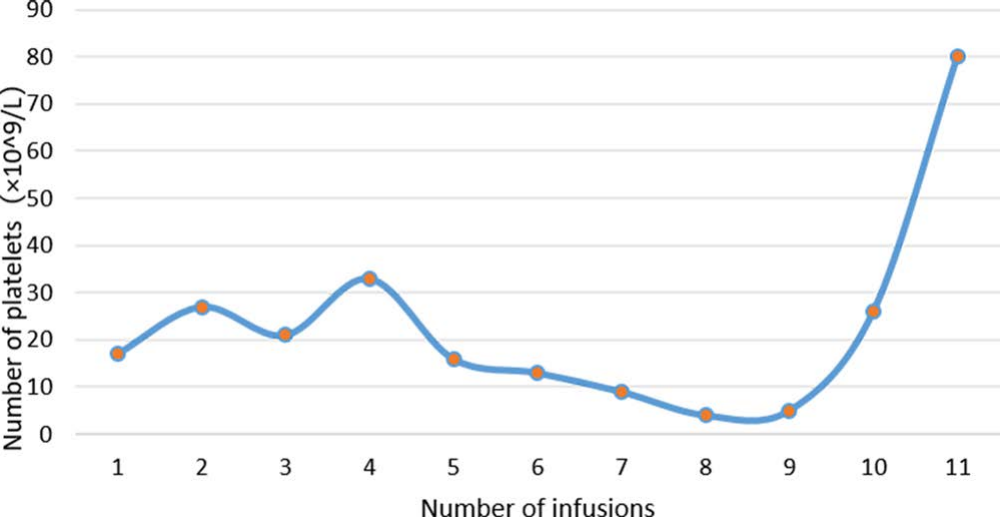
Platelet count of the patient after several transfusions.

The patient presented with severe refractory thrombocytopenia. Prior to any HLA/HPA-matched transfusion (i.e., before the 9th transfusion), the mean platelet count was recorded at (15.2 ± 3.8) × 10^9^/L (n = 8 measurements; range: (5–18) × 10^9^/L). This finding confirms an inadequate response to conventional unmatched platelet support in Table 5 (Fig. 4).

**Table 5:** Efficacy of HLA/HPA-matched platelet transfusions.

Transfusion no.	Donor HLA profile	Donor HPA profile	CCI	Platelet count (×10^9^/L)	Transfusion response
1–8	Unmatched	–	–	<20†	Refractory
9	*A*02:01*, *A*02:01*, *B*48:01*, *B*67:01*	HPA-5aa	13	40	Partial response
10	*A*02:01*, *A*33:03*, *B*52:01*, *B*58:01*	HPA-5aa	33	80	Significant response
11	Same as No. 10	HPA-5aa	–	>80	Sustained remission

## 4. Discussion

Most cases of PTR are attributed to nonimmune factors, however, immune factors still account for approximately 20% of instances.^[2]^ Considering the patient’s history of multiple pregnancies, there may be underlying immune factors at play.^[3]^ The presence of HLA and HPA antibodies has been confirmed through the PAK-Lx test. Anti-HLA-I and anti-HPA antibodies are the main causes of immune-related PTR.^[4]^ The positivity rate of platelet antibodies in patients is positively correlated with the number of platelet transfusions, especially in patients who solely rely on platelet transfusions. The patient is stimulated to produce complex platelet antibodies after multiple exposures to exogenous platelet antigens following transfusion.^[5]^ In addition to HLA-I antigens, heterozygosity of HPA-3 and HPA-15 systems, followed by HPA-2 and HPA-5 systems is commonly observed in the Chinese population. These antigens induce the production of antibodies, leading to the development of PTR.^[6–8]^

Here, the patient was a pregnant woman with AML. Despite multiple platelet transfusions, no increase in platelet count or improvement in the patient condition was observed. Subsequently, anti-HLA class I and anti-HPA-5b complex antibodies were observed in serum samples of the patient. The patient HLA-I genotypes were A * 30:01, A * 33:03, B * 13:02, and B * 44:03. Exposure to multiple human platelets resulted in the production of specific antibodies, such as anti-A2, anti-A24, anti-A68, anti-B15, and anti-B7 antibodies, and the positivity rate of PRA was 54%, indicating that the patient was in a highly sensitized state. This is consistent with the epidemiological characteristics of anti-HLA antibodies in haploid stem cell transplantation candidates.^[9]^

PAK-Lx, a test for platelet antibody detection and analysis, exhibits high throughput and sensitivity in the detection of the antibodies against different specific antigens, such as HLA-I, GPIIb/IIIa, GPIb/IX, GPIa/IIa, and CD36.^[10]^ Specificity of the anti-HLA-I antibody can be further evaluated by combining the anti-HLA-I antibody with the LIFECODES LSA Single Antigen Reagent. However, these methods are expensive, time-consuming, and require special instruments. Moreover, rapid detection capacity is limited, and platelet glycoprotein antigens are purified and coated on the surface of microbeads. Various HPA antigens, such as HPA-3 and HPA-5, act as mutant antigens. Extraction and purification can easily change the antigen conformation, resulting in the weakening of antigen–antibody reactions or omission of antibodies. Therefore, the results should be carefully analyzed. Moreover, fresh intact platelets should be used as antigens for antibody analysis.^[11,12]^

Antigens of the HPA-5 system are located in GPIa and encoded biallelically. Notably, 1000 to 2000 copies of α2 molecules are present on the surface of normal platelets, and each α2 molecule expresses a single epitope of HPA-5 antigen.^[13]^ Integrin α2 is widely distributed on the surface of various cells, but only platelet cells express the HPA-5 epitope, and no other cells exhibit antigenicity.^[14]^ In this study, anti-GPIa/IIa antibody was weakly positive in PAK-Lx samples, and HPA typing revealed HPA-5 as an aa homozygote. Combined with previous reports, we inferred that the sample contains the anti-HPA-5b antibody. To prevent the interference of HLA antibodies in the patient sample, O-type platelets of HPA-5aa and HPA-5ab treated with chloroquine diphosphate were used as antigens for detection and analysis via solid-phase immunoassay, as chloroquine diphosphate only destroys the structure of the HLA glycoprotein without affecting the antigenicity of other platelet glycoproteins; anti-HPA-5b antibody presence in the sample was subsequently confirmed.^[15,16]^ One limitation of this method is that due to the distribution frequency of antigen genes, type O platelets with HPA-5bb may not be screened in the early stage, thus the HPA 5ab platelet test may weaken the anti-HPA-5b antibody reaction in the sample.

In this study, various methods were used to comprehensively analyze the specificity of anti-platelet antibodies in the samples of patients with ineffective platelet transfusion, thus providing a reference for the clinical diagnosis and treatment of PTR.

## Acknowledgments

We thank Jiangsu Weihe Biotechnology Inc. for guidance and help in our experimental technology.

## Author contributions

**Conceptualization:** Yiming Jin, Mingyuan Wang.

**Data curation:** Yihan Wang.

**Formal analysis:** Shengbao Duan, Hongmei Wang, Longhai Tang.

**Investigation:** Yihan Wang, Yujue Wang, Longhai Tang.

**Methodology:** Zhen Liu, Shengbao Duan.

**Project administration:** Mingyuan Wang.

**Resources:** Zhen Liu, Hongmei Wang.

**Software:** Chunyan Lin.

**Supervision:** Yujue Wang, Mingyuan Wang.

**Validation:** Chunyan Lin, Yiming Jin.

**Writing – original draft:** Zhen Liu, Shengbao Duan, Chunyan Lin, Yiming Jin, Hongmei Wang, Yujue Wang, Longhai Tang, Mingyuan Wang.

**Writing – review & editing:** Yihan Wang, Zhen Liu, Shengbao Duan, Chunyan Lin, Yiming Jin, Hongmei Wang, Yujue Wang, Longhai Tang, Mingyuan Wang.
